# Estimating dengue incidence and hospitalization in Malaysia, 2001 to 2013

**DOI:** 10.1186/s12889-018-5849-z

**Published:** 2018-08-02

**Authors:** Yuan Liang Woon, Chee Peng Hor, Keng Yee Lee, Siti Fatimah Zahra Mohd Anuar, Rose Nani Mudin, Mohd Khadzir Sheikh Ahmad, Suhaya Komari, Faridah Amin, Rahman Jamal, Wei Seng Chen, Pik Pin Goh, Lena Yeap, Zhuo Ren Lim, Teck Onn Lim

**Affiliations:** 1 Clinical Research Centre, Ministry of Health Malaysia, c/o Third Floor, Dermatology Block, Hospital Kuala Lumpur, Jalan Pahang, 50586 Kuala Lumpur, Malaysia; 2 Kepala Batas Hospital, Ministry of Health Malaysia, Jalan Bertam 2, 13200 Kepala Batas, Penang, Malaysia; 3 Sector of Vector-Borne Disease, Disease Control Division, Ministry of Health Malaysia, 62590 Putrajaya, Malaysia; 4 Health Informatic Centre, Planning Division, Ministry of Health Malaysia, 62590 Putrajaya, Malaysia; 5 National Public Health Laboratory, Lot 1853, Kg, Melayu, 47000, Sungai Buloh, Selangor Malaysia; 6 UKM Medical Molecular Biology Institute, UKM Medical Centre, Jalan Yaacob Latiff, Bandar Tun Razak, 56000 Cheras, Kuala Lumpur, Malaysia; 7 Klinik Alam Medic, 41, Jalan Perdana 3/4, Taman Puchong Perdana, 47100 Puchong, Selangor Malaysia; 8 Stats Consulting Pte Ltd, D7-3-1, Block D7, Pusat Perdagangan Dana 1, Jalan PJU 1A/46, PJU 1A, 47301 Petaling Jaya, Selangor Malaysia; 9 ClinResearch Pte Ltd, D7-3-1, Block D7, Pusat Perdagangan Dana 1, Jalan PJU 1A/46, PJU 1A, 47301 Petaling Jaya, Selangor Malaysia

**Keywords:** Dengue, Incidence, Notification, Hospitalization, Time trend, Malaysia

## Abstract

**Background:**

Epidemiologic measures of the dengue burden such as prevalence and incidence are important for policy-making and monitoring the progress of disease control. It is a common practice where epidemiologic and economic research estimate dengue burden based on notification data. However, a basic challenge in estimating the incidence of dengue is that a significant proportion of infected population are asymptomatic. It can be overcome by using mathematical models that relate observed prevalence and mortality to incidence. In this study, we estimate the trend of dengue incidence and hospitalization in Malaysia.

**Methods:**

This study is based entirely on the available secondary data sources on dengue in Malaysia. The age-specific incidence of dengue between 2001 and 2013 was estimated using the prevalence and mortality estimates in an incidence-prevalence-mortality (IPM) model. Data on dengue prevalence were extracted from six sero-surveys conducted in Malaysia between 2001 and 2013; while statistics on dengue notification and Case Fatality Rate were derived from National Dengue Surveillance System. Dengue hospitalization data for the years 2009 to 2013 were extracted from the Health Informatics Centre and the volumes of dengue hospitalization for hospitals with missing data were estimated with Poisson models.

**Results:**

The dengue incidence in Malaysia varied from 69.9 to 93.4 per 1000 population (pkp) between 2001 and 2013.The temporal trend in incidence rate was decreasing since 2001. It has been reducing at an average rate of 2.57 pkp per year from 2001 to 2013 (*p* = 0.011). The age-specific incidence of dengue decreased steadily with dengue incidence reaching zero by age > 70 years. Dengue notification rate has remained stable since 2001 and the number of notified cases each year was only a small fraction of the incident cases (0.7 to 2.3%). Similarly, the dengue hospitalization was larger but still a small fraction of the incident cases (3.0 to 5.6%).

**Conclusion:**

Dengue incidence can be estimated with the use of sero-prevalence surveys and mortality data. This study highlights a reducing trend of dengue incidence in Malaysia and demonstrates the discrepancy between true dengue disease burden and cases reported by national surveillance system. Sero-prevalence studies with representative samples should be conducted regularly to allow better estimation of dengue burden in Malaysia.

## Background

Dengue has become a global public health concern. Epidemiologic measures of the burden of dengue such as its prevalence and incidence, by age and over time, are important for policy-making and monitoring the progress of disease control. World Health Organization (WHO) reported the global incidence of dengue has increased by 30-fold in the past 50 years and estimated some 50 to 100 million new infections occurred annually, with approximately 20,000 deaths [1]. A more recent estimate using the cartographic approach has increased this number up to 390 million infections a year, more than three times WHO’s estimate. Asia bore a disproportionate 70% of the global burden [2]. The national dengue surveillance system is widely used as a proxy measure to report (or estimate) dengue incidence [3–13]. This is in contrast to other major infectious diseases such as human immunodeficiency virus (HIV), tuberculosis and malaria, where most of the affected countries routinely estimate these epidemiologic measures [14–16].

A challenge in estimating the incidence of dengue is that a significant proportion of infected people are asymptomatic and these cases are not captured by passive surveillance system. As a result, symptomatic or treated cases, or cases notified to the national surveillance system underestimate disease incidence [17]. A method to overcome this challenge is to use mathematical models that relate observed prevalence and mortality to incidence. Sero-prevalence data which identify both asymptomatic and symptomatic past infections, is the crucial data source for such models. This method is widely used to estimate the incidence of HIV [18], tuberculosis [15] and malaria [19], but apparently rarely so for dengue.

In this study, we estimated the trend of dengue incidence in Malaysia based on six sero-prevalence surveys between 2001 and 2013, and e-Dengue registry. We also estimated the dengue hospitalization rates based on national hospital discharges database.

## Methods

This study is based entirely on the available secondary data sources on dengue in Malaysia. Data on Malaysian population were obtained from the Department of Statistics (DOS) [20, 21]. The Medical and Research Ethics Committee (MREC) from Ministry of Health (MOH) approved the study (NMRR-16-2301-33,463).

### Data source: sero-prevalence studies

**Fig. 1:**
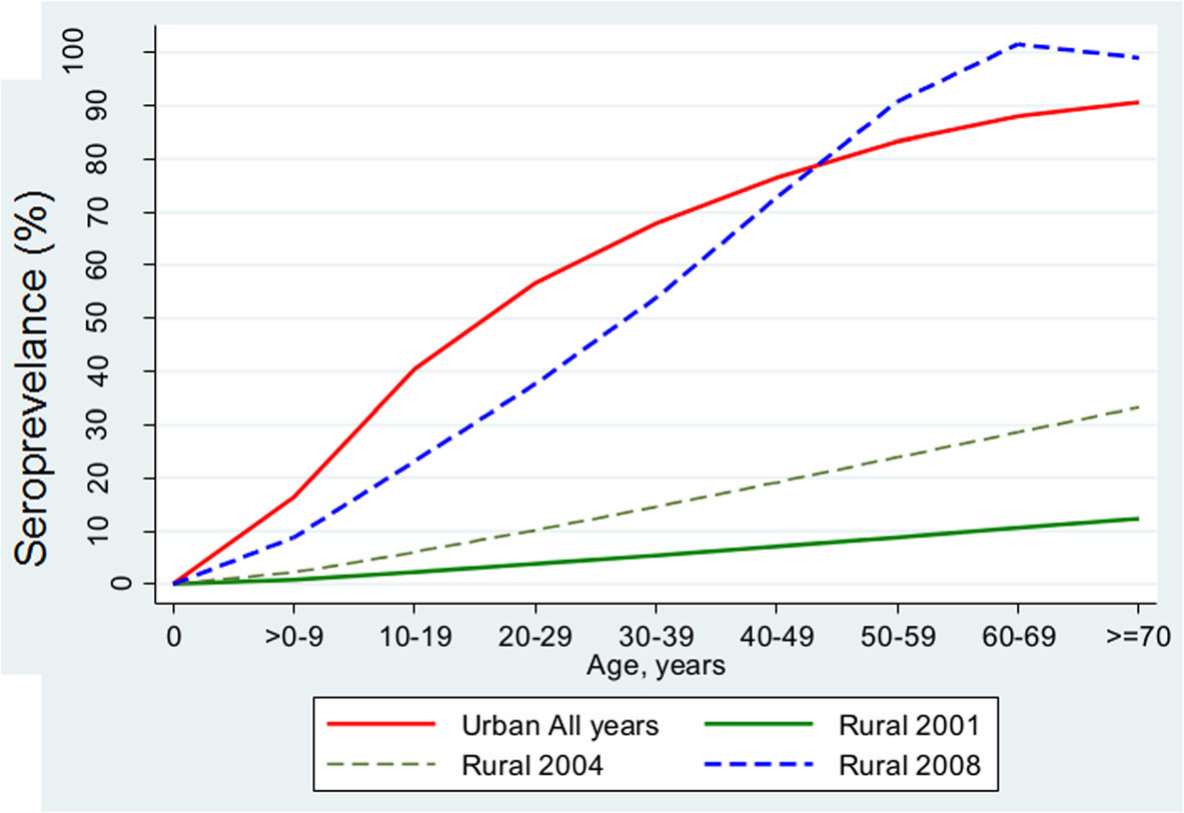
Age-specific dengue sero-prevalence by urban-rural locations, Malaysia, 2001–2013. Reprinted from “Rural-urban Comparisons of Dengue Seroprevalence in Malaysia” by Chew CH, Woon YL, Amin F, et al., BMC Public Health, 16(1), 8. [55]

Data on prevalence of dengue were extracted from six sero-surveys conducted in Malaysia between 2001 and 2013, which comprised five urban and two rural series. The serological tests used in all surveys were dengue IgG indirect enzyme-linked immunosorbent assay (ELISA), which neither distinguish between the four dengue serotypes, nor between primary and secondary infections. Sero-positivity on the ELISA test therefore identified past primary infection by any of the four serotypes. Data from the six surveys were pooled to estimate the age-specific sero-prevalence rates between the period 2001 and 2013, which has been reported elsewhere [22]. Figure 1 shows the age-specific dengue sero-prevalence estimates reproduced from that study. The dengue sero-prevalence was constant in urban areas for all years, while dengue sero-prevalence in rural areas was rising and converged with urban sero-prevalence by 2008.

### Data source: dengue notification

We obtained dengue notification data for the years 2001–2013 from National Dengue Surveillance System, which all dengue cases encountered by all healthcare facilities including laboratories in the country must be reported to by law. Statistics on Case Fatality Rate (CFR) of dengue were derived from this source [23].

### Data source: hospitalization due to dengue

We extracted dengue hospitalization data for the years 2009 to 2013 from the Health Informatic Centre (HIC) of the Ministry of Health. The centre maintains a data warehouse containing data on hospital discharges from both public and private hospitals in Malaysia. We identified all cases of dengue based on International Classification of Diseases, Tenth Revision, Clinical Modification (ICD-10-CM) codes. Records of patients with the following codes were included: A90 for dengue fever (classical dengue) and A91 for dengue haemorrhagic fever. We also obtained data on dengue hospitalization for years 2010 through 2013 from four private insurance companies which combined 90% market share in Malaysia, in order to validate the model for estimating dengue hospitalization.

### Statistical methods

**Fig. 2:**
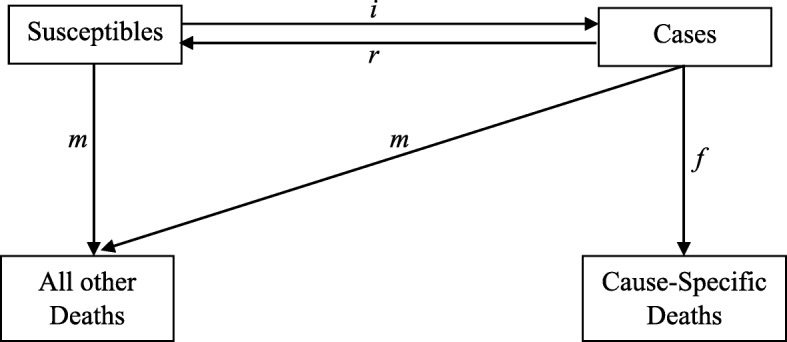
Conceptual Framework for Incidence-Prevalence-Mortality Model

We estimated the age-specific incidence of dengue between 2001 and 2013 by combining the prevalence and mortality estimates in the incidence-prevalence-mortality (IPM) model [24]. This model is based on the conceptual framework illustrated in Fig. 2 below [24].

In brief, this model describes a population being in different states, and the transition hazards determine how individuals move from one state to another. Within a population, an individual can be either susceptible to or affected by the disease. In this study, the susceptible individuals can get infected by DENV at rate *i*; while individuals infected by DENV may recover from the infection at rate *r* or die from dengue-specific mortality at rate *f*. In addition, both groups of individuals (susceptible and case) are at risk of dying from other causes at rate *m*. This model has four transition hazards: incidence, case-fatality, all other mortality and recovery. Under the assumption of a steady-state situation in IPM model, time is equivalent to patient’s age in this study. Therefore, a set of linear differential equations can be defined to characterize transition between the states shown in Fig. 2. In this case, the recovery transition hazard will be zero, because none of the dengue sero-positive cases would be reversed into dengue naïve state. The equations used in this estimation are shown as below:

$$ \frac{dS_{age}}{dt_{ij}}=-{i}_{ij}\ast {S}_{ij}+{r}_{ij}\ast {C}_{ij}-{m}_{ij}\ast {S}_{ij} $$

$$ \frac{dC_{age}}{d\ {dt}_{ij}}=-{f}_{ij}\ast {C}_{ij}+{i}_{ij}\ast {S}_{ij}-{m}_{ij}\ast {C}_{ij} $$

$$ \frac{dD_{age}}{d\ {dt}_{ij}}={f}_{ij}\ast {C}_{ij}+{m}_{ij}\ast {C}_{ij}+{m}_{ij}\ast {S}_{ij} $$

Where

S_ij_ is the number susceptible persons at year i in age group j

_Cij_ is the number dengue cases at year i in age group j

S_ij_ is the number deaths at year i in age group j

i_ij_ is the dengue incidence rate at year i in age group j

r_ij_ is the recovery rate from dengue at year i in age group j, which is zero for sero-positive status.

f_ij_ is the dengue specific mortality rate at year i in age group j

m_ij_ is the population general mortality rate at year i in age group j

We also assumed a constant mortality rate across all age groups according to the CFR of the respective year. The model took into account the differences in dengue prevalence between urban and rural areas. However, data on rural dengue prevalence were available only up to year 2008 with an age-standardized prevalence 43%, compare with urban prevalence of 54%. We therefore assumed there was no change in the rural prevalence of dengue after 2008 in our model. Sensitivity analyses were performed to evaluate uncertainty due to this assumption. The range of plausible values were ± 20% of the assumed rural prevalence after 2008.

Availability of dengue hospitalization data varied by year, from 160 out of total of 371 hospitals in 2009 to 160 out of total of 394 hospitals in 2013. To estimate dengue hospitalization rates, we used a Poisson model for cross-sectional time series data and generalised estimating equation to estimate the volume of dengue hospitalization for hospitals with missing data. Poisson regression was used because the dependent variable (number of hospital discharge for dengue) is an observed count. The model included characteristics of the geographical location (district) where a hospital is located (total hospitalization rate per 1000 population, standardised mortality ratio, percentage of population aged over 65 and under 5 years) and hospital level characteristics (public or private hospital, type of hospital (general, specialty or maternity), and bed capacity). The model was validated and calibrated by assessing the consistency between the observed and model-predicted number of dengue hospitalizations. We also externally validated the model by comparing its estimate of dengue hospitalization rates against independent estimates using data from private health insurance (PHI). Estimates of dengue hospitalization rates from PHI data were 3.0, 1.8, 1.6, and 4.0 per 1000 population (pkp) for years 2010 through to 2013. These were comparable though lower than estimates from the above modelling of hospitals discharge data, which are 4.0, 2.6, 2.1 and 3.6 pkp for the same years. These two sets of estimates were not similar because the population base for the first estimate was the privately insured population while the second was entire population of Malaysia.

## Results

**Fig. 3:**
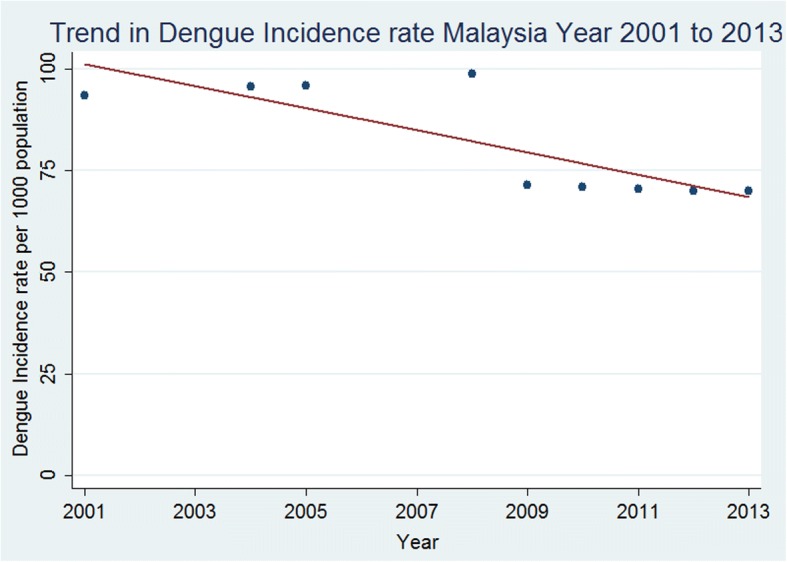
Trend in dengue incidence rate per 1000 population, Malaysia, year 2001 to 2013

**Fig. 4:**
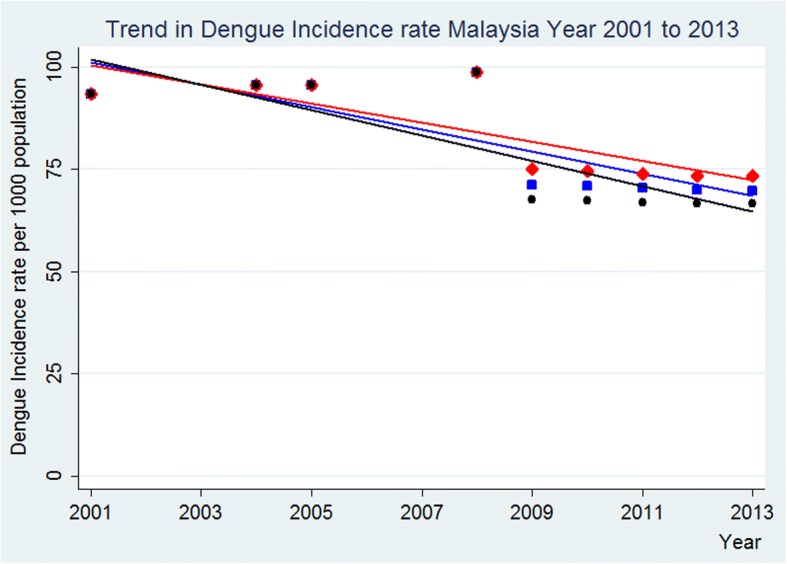
Results of sensitivity analysis using ±20% than the assumed rural prevalence rates after 2008 for the trend in dengue incidence rate per 1000 population, Malaysia, year 2001 to 2013

The number of people infected by dengue in Malaysia varied from approximately 2.2 million in 2001 to 2.1 million in 2013, representing an annual incidence ranging between 69.9 and 93.4 pkp (Table 1). These estimates translate to, between 7 and 9% of the population were infected by dengue each year between 2001 and 2013. While these rates were high, the temporal trend in incidence rate was decreasing since 2001. It has been reducing at an average rate of 2.57 pkp per year (95% CI: -4.53, − 0.61) from 2001 to 2013 (Mann-Kendall trend test, *p* = 0.011) (Fig. 3). When the assumed rural prevalence of dengue after 2008 were changed by ±20%, the dengue incidence has changed only ±4.8% in 2013 and the declining trends in incidence rate are still obvious (Fig. 4).

**Table 1:** Trends in Dengue Incidence, Prevalence, Notification and Hospitalization, Malaysia 2001 to 2013

Year	Population, 000’	Estimated number of people infected with dengue	Cumulative risk of dengue by age 70+ (%)	Incidence of dengue (pkp*)	Number of notified dengue cases	Notification rate of dengue (pkp*)	Number of hospital discharges for dengue	Hospitalization rate for dengue (pkp*)
2001	24,030.5	2,244,920	100	93.4	16,368	0.68	–	–
2002	24,542.5	–	–	–	32,767	1.34	–	–
2003	25,038.1	–	–	–	31,545	1.26	–	–
2004	25,541.5	2,445,835	100	95.6	33,895	1.33	–	–
2005	26,045.5	2,493,365	100	95.7	39,654	1.52	–	–
2006	26,549.9	–	–	–	38,556	1.45	–	–
2007	27,058.4	–	–	–	48,846	1.81	–	–
2008	27,567.6	2,772,889	100	98.8	49,335	1.79	–	–
2009	28,081.5	2,002,751	88	71.3	41,486	1.48	80,797	2.87
2010	28,588.6	2,028,228	88	70.9	46,171	1.62	113,382	3.96
2011	29,062.0	2,049,302	88	70.5	19,884	0.68	76,723	2.64
2012	29,510.0	2,064,400	87	69.9	21,900	0.74	61,463	2.08
2013	29,915.3	2,092,312	88	69.9	43,346	1.45	106,884	3.57

*pkp: per 1000 population

**Fig. 5:**
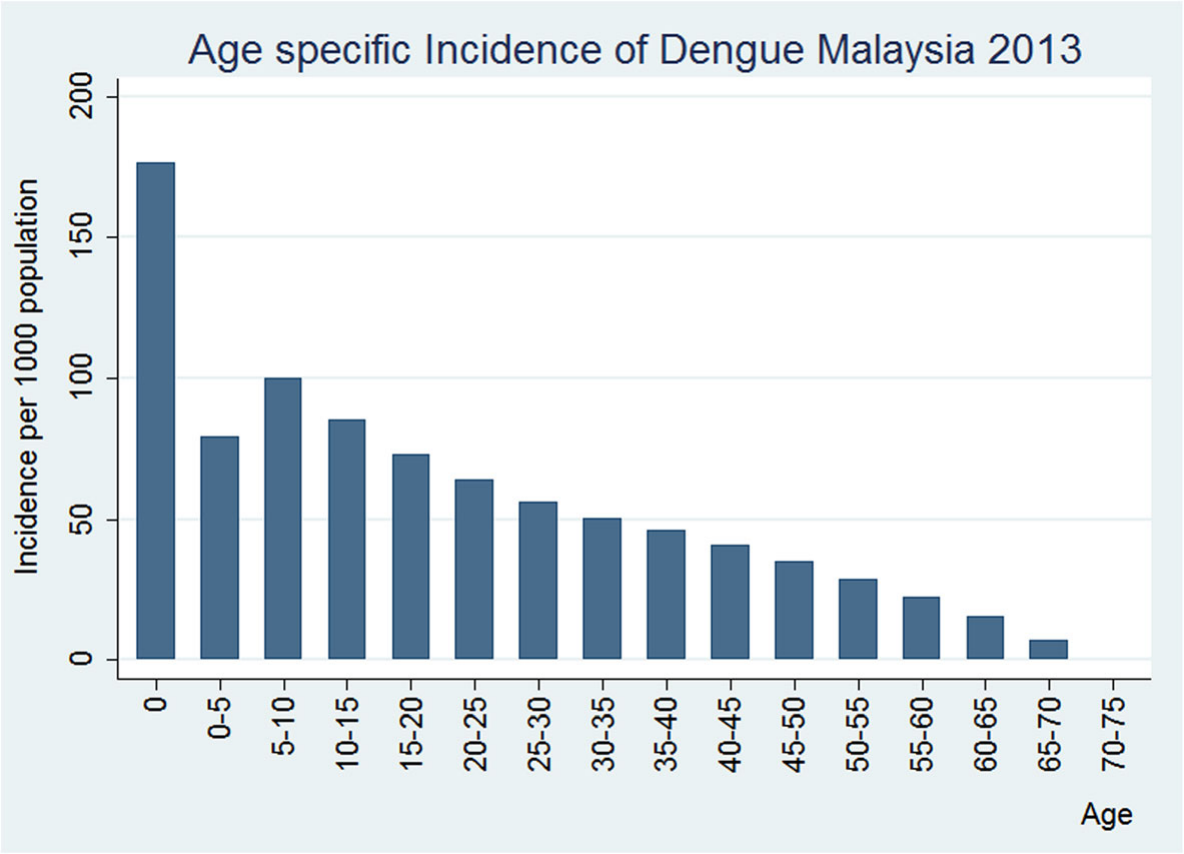
Age-specific dengue incidence rate per 1000 population, Malaysia, Year 2013

**Fig. 6:**
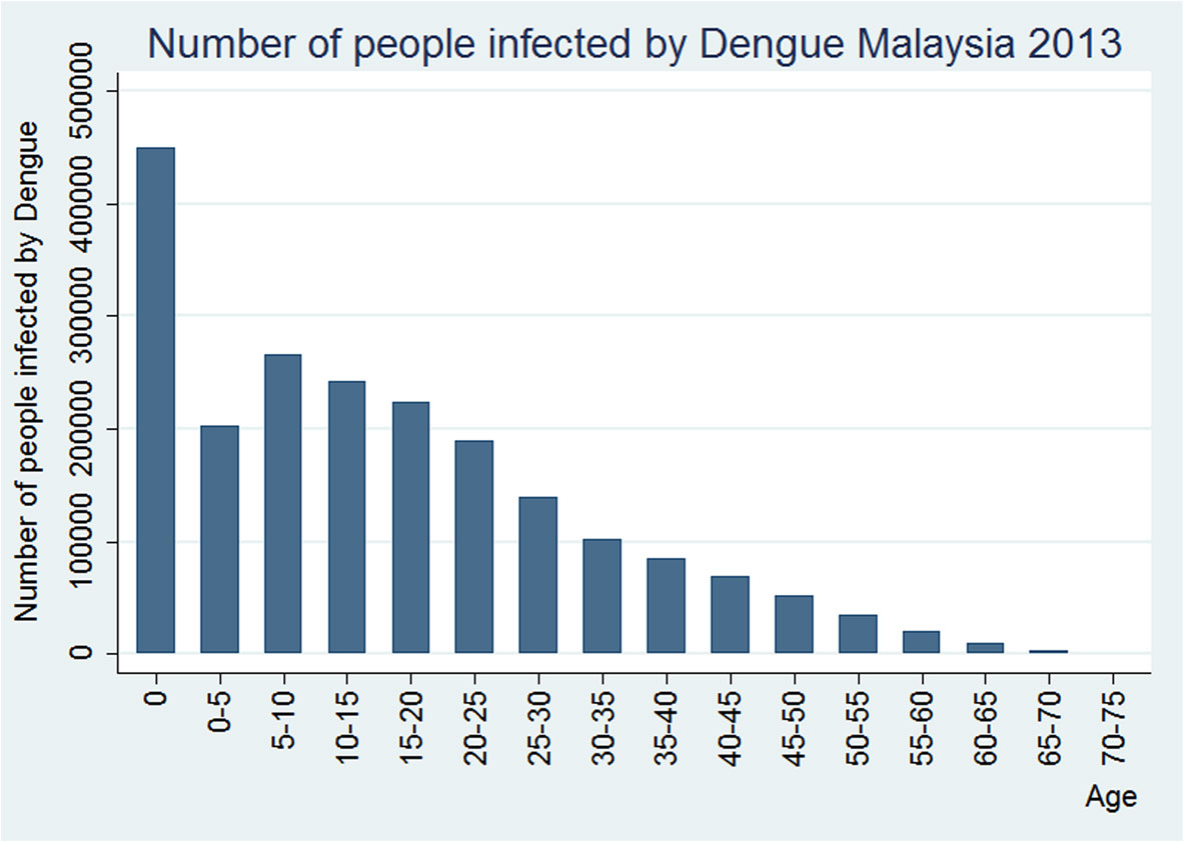
Number of people infected by dengue by age, Malaysia, Year 2013

**Fig. 7:**
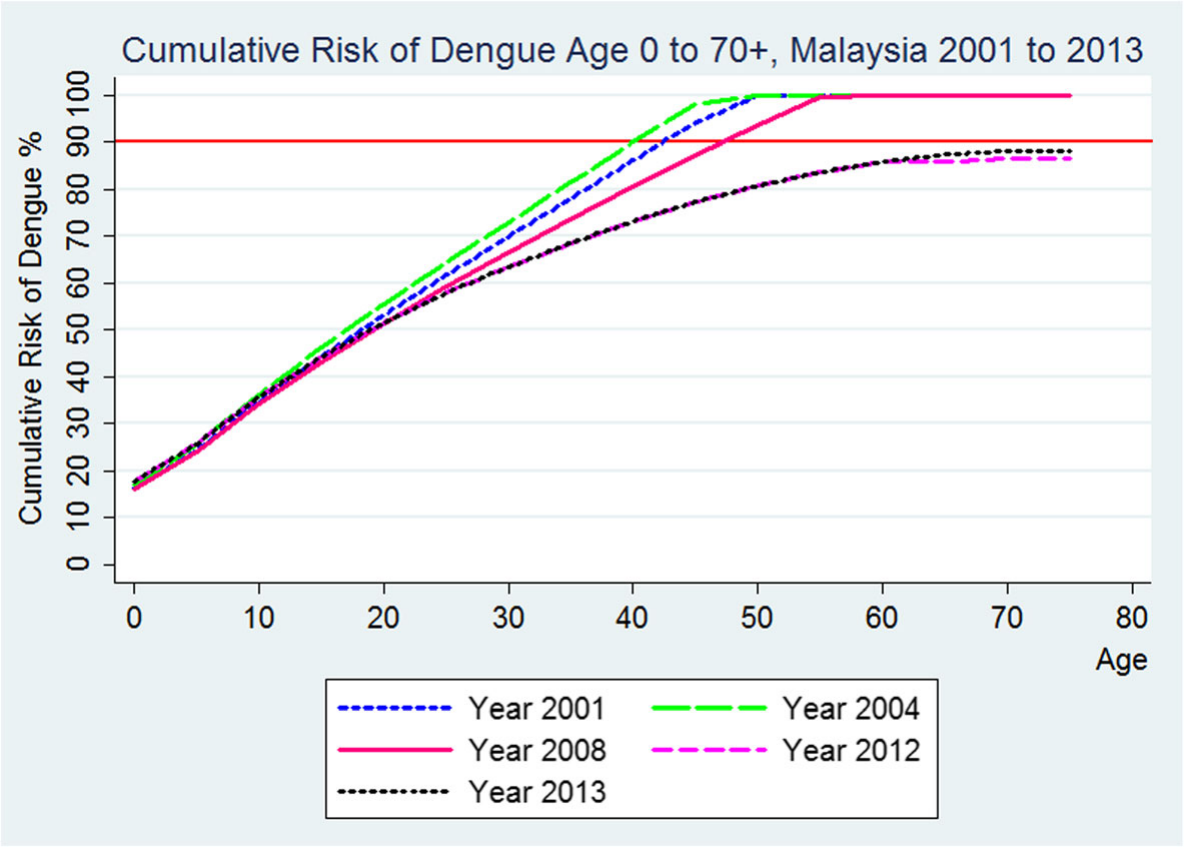
Cumulative risk of dengue from age 0 to 70+ in Malaysia, 2001 to 2013

The incidence rate for the age group 0 to 4 years was 176.6 pkp in 2013, thereafter the age-specific incidence of dengue decreased steadily with dengue incidence to reach zero by age > 75 years (Fig. 5). This translates to about 450,000 of children were infected between age 0 and 4 years, while another half a million were infected by age 15 years (Fig. 6). At such high incidence rates at early ages, the cumulative risk of being infected by dengue had reached 100% by age 60 (Fig. 7). However, the cumulative risk of infection by age 70+ has decreased to 88% in 2013, reflecting the declining temporal trend in dengue incidence (Fig. 7).

Results on dengue notification and hospitalization rates are presented in Table 1 for comparison with the incidence rate. The number of dengue infections notified to the authority each year was a small fraction of the incident cases (0.7 to 2.3%) even though notified dengue cases would have included a large but unknown number of secondary and tertiary dengue infections. Dengue notification rate has remained stable since 2001. The number of people admitted into hospitals each year because of dengue was also a larger but still a small fraction of the incident cases (3.0 to 5.6%). Dengue hospitalization rate appeared to be decreasing slightly over time.

## Discussion

This study attempts to estimate the national dengue incidence and hospitalization rates as measures of the disease burden in Malaysia. Assessment of dengue disease burden have been carried out in various settings using different approaches. While observational cohort studies are preferred to estimate dengue incidence in a small local area, for national estimate, an alternative approach is to use serial sero-prevalence data with an IPM model. To the authors’ knowledge, this is the first study which estimated dengue incidence by IPM model.

### Dengue incidence and hospitalizations estimates using notification data

Dengue is a notifiable infectious disease in Malaysia, according to the Prevention and Control of Infectious Diseases Act 1988. One of the common approaches was to estimate the dengue incidence by utilizing notification data collected through a national surveillance system [3, 5, 7, 8, 11, 12, 25–29]. While the WHO and other studies estimated Malaysia dengue incidence between 2009 and 2010 as 1.5–1.7 pkp by utilizing notification data [3, 7, 8, 11, 12, 29], our study using an IPM approach demonstrated the dengue incidence to be at least 40 times higher than what had been notified. Under-reporting of cases is a recognized limitation for routine passive surveillance systems [5, 27, 28, 30–36], as these systems are usually designed to detect outbreaks instead of estimating the disease burden. This explained why our estimates are higher than studies which estimated dengue incidence solely based on dengue notification data. In addition, the estimated dengue hospitalization rates were also higher than the notification rates captured by the national surveillance system although all dengue cases treated in hospital should have been notified and reported to the authority. These findings demonstrated substantial underreporting of dengue fever and that official statistics underestimate true incidence rate, which concurs with another study estimating the dengue burden in Southeast Asia [37]. The study by Nealon J et al. had also observed a large proportion of symptomatic dengue cases in Malaysia were not captured by national surveillance systems and this gave rise to a high expansion factor of 31.7 employed in their model [32]. An expansion factor is referring to the number by which the reported cases need to be multiplied in order to get the most accurate estimate of the true number of episodes. Under-reporting of dengue cases by national surveillance system has also been observed in Indonesia, Thailand, Singapore, Vietnam, Brazil and Cambodia [5, 27, 28, 32–37]. These results underscore the lack of reliability of using notification rate as a source of data to estimate dengue incidence. Nevertheless, this is because notification data are more readily available while sero-prevalence data are limited. Almost all health policy analyses [1, 38–41] and epidemiologic and economic research on dengue published to date [37, 42, 43] based their estimates of disease burden on notification data. Improvement in the estimation of dengue incidence would allow better comparison of dengue burden across countries, while guiding refined planning for dengue control programs.

The dengue incident rate decreased at a mean rate of 2.57 pkp annually from 2001 to 2013. The dengue sero-prevalence in urban areas over the same period was constant; meanwhile, the dengue prevalence in rural areas in the country had increased since 2001 and converged with levels observed among urban population by year 2008 [22]. Data on rural dengue prevalence were available only up to year 2008, and therefore we assumed no or minimal change in rural prevalence after 2008 which was supported by the sensitivity analyses. We postulated there is a reduction in pool of susceptible populations in both urban and rural areas which could be attributable to the fairly constant sero-prevalence with a population growth and multimodal public heath preventive strategies.

### Dengue incidence estimates: Cohort study versus sero-prevalence data with IPM model

Prospective cohort study is the best approach in estimating the true dengue incidence. However, it is resource intensive, time consuming and practically challenging to be conducted on nation-wide scale. To date, cohort studies have been conducted in Thailand [34, 44, 45], Indonesia [33, 44], Vietnam [36, 44, 46], Philippines [44], Cambodia [47], Nicaragua [35, 48] and Latin America [49] to assess dengue incidence in selected locations within those countries. We summarize these incidence estimates from these cohort studies to compare with our estimates using the IPM approach in Table 2.

**Table 2:** Comparison of Incidence Estimates by IPM Approach with other Cohort Studies

**#**	Area, Country	Author [Ref]	Year	Age in years	Incidence of dengue (%)	Incidence of symptomatic dengue (%)	Incidence of hospitalized dengue (%)
1.	Malaysia	Present study	2009	All	7.13	n/a	0.29
	Malaysia		2009	0–15	10.7	n/a	0.22
	Malaysia		2013	All	6.99	n/a	0.36
	Malaysia		2013	0–15	11.0	n/a	0.3
2.	Rayong, Thailand	Sangakawibha [56]	1980–1981	4–14	9.4	n/a	0.1
3.	Bangkok, Thailand	Burke [51]	1980–1981	4–16	11.8	0.7	0.4
4.	Yangon, Myamar	Thein [57]	1984–1988	1–9	5.1	n/a	0.3
5.	Yogyakarta, Indonesia	Graham [58]	1996–1996	4–9	29.2	0.6	0.4
6.	Kamphaeng Phet Thailand	Endy [45, 59]	1998–2002	7–11	7.3	3.9	1.0
7.	Iquitos Peru	Morrison [60]	1999–2005	5–20	34.5	n/a	n/a
8.	W Java Indonesia	Porter [33]	2000–2002	18–66	7.4	1.8	0.1
9.	Managua, Nicaragua	Balmaseda [61]	2001–2002	4–16	9.0	0.85	n/a
10.	Maracay Venezuela	Comach [62]	2001–2002	5–13	16.9	n/a	n/a
11.	Kamphaeng Phet Thailand	Mammen [63]	2004–2006	4–13	6.7	2.2	0.5
12.	Ratchaburi Thailand	Sirivichayakul [64], Sabchareon [34]	2006–2009	3–11	3.6	3.6	1.6
13.	Managua, Nicaragua	Balmaseda [48]	2004–2010	2–9	9.0	0.85	n/a
14.	Long Xuyen Vietnam	Tien [36]	2004–2007	2–15	3.0	3.0	1.2
15.	Malaysia	L’Azou [50], Nealon [32]	2010	2–16 (participants in control arm of vaccine trial)	n/a	Overall: 2.2 Age 2–4: 2.5 Age 5–8: 1.7 Age 9–12: 3.1 Age 13–16: 1.7	0.85

Nearly all of these studies were conducted among pediatric population, except the one study by Porter KR et al. which determined the dengue incidence among adults aged 18–66 years old [33]. All studies included follow-ups to identify febrile patients for serological and/or molecular testing for dengue infection. L’Azou et al. and Nealon J et al. in their recent dengue vaccine trial reported that dengue incidence among Malaysian children aged 2–14 years in the control arm of the study was 2.05% (95% CI 1.10, 3.72) [32, 50]. Our findings in this study are consistent with other published estimates. Most dengue cases are asymptomatic [2, 51, 52] but our analysis takes into consideration both symptomatic and asymptomatic cases and thus produces higher estimates in Malaysia than previous studies [3, 7, 8, 11, 12, 29]. The difference of incidence estimates between our study and the other published studies may also be subject to the variation in the year of study conducted, geographical location and methodological approaches.

### Hospitalized dengue in Malaysia

Hospitalization due to an acute illness like dengue is a standard measure of morbidity. Dengue hospitalization in particular is also the critical driver of economic cost of dengue to society. On top of that, hospitalization also leads to loss in economic productivity arising from sick workers taking sick leave or parents taking leaves to care for their sick children. There are limited published estimates of dengue hospitalization. Economic research on dengue hospitalization largely depends on notified cases, which could be obscure and uncertain [53]. Our estimated hospitalization episodes in 2009 and 2010 were 80,797 and 113,382, respectively. These estimates were slightly higher than the estimate reported by Shepard DS et al., which was 62,256 episodes (95% CI 42,561, 108,311) [10]. On the other hand, L’Azou M et al. and Nealon J et al. utilized data collected from dengue vaccination trial and estimated the incidence of dengue hospitalization among Malaysians aged 2–16 years to be 0.85% [32, 50]. This estimation is much higher than ours which ranged between 0.22 and 0.36%. We postulated this discrepancy is attributable to the nature of the clinical trials, where the subjects are closely followed up, and necessary action is taken when there is any reported adverse event. Our observation that there was a reducing trend of hospitalization rate could also be a reflection of the reducing trend of dengue incidence rate across the years.

### Study limitations

We acknowledged that the sero-prevalence surveys were conducted for a different purpose and the data were not representative of the population. Although potential sampling bias might be introduced in the surveys, use of existing sero-prevalence data sources helps to increase the data volume. In view of limited data availability and high percentage of asymptomatic dengue cases, this is the only feasible way for us to obtain the dengue prevalence of previous years. Moreover, sero-prevalence data represented history of past dengue infections, and was unable to differentiate secondary from primary infections. Another limitation was the assumption of recovery transition hazards being zero, based on the sero-status of each individual, as none of dengue sero-positive individual would revert to dengue-naïve state. We assumed a constant mortality rate across all age groups, and the overall dengue CFR was applied for all ages for age-specific mortality rate. This is acceptable given that the CFR of dengue infection in Malaysia has been low and remains constant at about 0.2–0.3 per 100,000 population over the study period [23]. Furthermore, only about a quarter of Malaysians have PHI coverage [54], but this was the best available data for us to validate the model for estimating dengue hospitalization in the country.

## Conclusion

In conclusion, this study highlights a decline in dengue incidence in Malaysia between 2001 and 2013, and demonstrates the discrepancy between true dengue disease burden and cases reported by national surveillance system in Malaysia. In addition, this study also demonstrated dengue incidence can be estimated with the use of IPM model. A well designed sero-prevalence study with representative samples should be conducted regularly in order to give better estimates of the dengue burden in Malaysia. This information is useful in monitoring the progress of national dengue epidemics and guiding future dengue control and prevention program.

## Abbreviations

CFR: Case fatality rate
DOS: Department of Statistics
ELISA: Enzyme-linked immunosorbent assay
HIC: Health Informatic Centre
HIV: Human immunodeficiency virus
ICD-10-CM: International classification of diseases, tenth revision, clinical modification
IPM: Incidence-prevalence-mortality model
MOH: Ministry of Health
MREC: Medical and Research Ethics Committee
PHI: Private health insurance
pkp: Per 1000 population
WHO: World Health Organization
